# Editorial: Epigenetic Reprogramming and Cancer Development Volume II

**DOI:** 10.3389/fcell.2021.823503

**Published:** 2022-01-05

**Authors:** Isidro Sánchez-García, Carolina Vicente-Dueñas, Geoffrey Brown

**Affiliations:** ^1^ Experimental Therapeutics and Translational Oncology Program, Instituto de Biología Molecular y Celular Del Cáncer, CSIC/Universidad de Salamanca, Salamanca, Spain; ^2^ Institute of Biomedical Research of Salamanca (IBSAL), Salamanca, Spain; ^3^ School of Biomedical Sciences, Institute of Clinical Sciences, University of Birmingham, Birmingham, United Kingdom

**Keywords:** epigenetics, cancer, stem cells, oncogenes, leukemia

From studies of acute myeloid leukemia (AML) in 2008, the cancer stem cell theory states that most, if not all, cancers arise via an oncogene-driven transformation of a stem/progenitor cell that gives rise to the range of cell types within a tissue. Leukemia stem cells (LSCs) sustain AML by giving rise to a hierarchy of cells that only partially differentiate. Additionally, some oncogenes appear to restrict hematopoietic stem cells (HSCs) to just one cell lineage. Restricting the expression of the oncogenes BCR-ABLp190, LMO2 and BCR-ABLp210 to HSCs, in transgenic mice *via* the Sca-1 promotor, caused B-cell acute lymphoblastic leukemia (B-ALL), T-cell acute lymphoblastic leukemia, and chronic myeloid (neutrophils) leukemia, in the respective mice. By contrast, it is considered that HSCs replenish the functional cell types to meet an organisms’ changing demands, even to the extent that they can divert from their initial chosen pathway towards a different type of mature cell.

From studies of identical twins that both developed childhood B-ALL at least two oncogenic insults are needed for leukemia. The *TEL/AML1* fusion gene (known also as *ETV6-RUNX1*) is the most common acquired genetic lesion in childhood B-ALL, and almost exclusively associated with this leukemia. *TEL/AML1* pre-leukemic clones are found frequently in neonatal blood, with few carriers developing disease. For the twins that both developed B-ALL, there is intra-placental sharing of a pre-leukemic clone with the same *TEL* and *AML1* breakpoints, and a further postnatal insult(s) is needed because the disease occurrence in the twins is asynchronous. Rodriguez-Hernández et al. have examined how *ETV6-RUNX1* together with another hit determine the lineage fate of the leukemia cells in childhood B-ALL. This leukemia seems to arise in an HSC because patients’ HSC-like cells that lack B-cell markers cause the human disease in mice. Rodriguez-Hernández et al. have shown that the second hit determines the B-cell fate of ETV6-RUNX1 B-ALL, and that *ETV6-RUNX1* can trigger both B- and T-cell leukemias. For childhood B-ALL, both hits have to occur in a cell that is not B-cell committed. In others word, B-ALL is triggered at the HSC/progenitor cell stage in a cooperative manner. A current view is that the second hit is triggered by exposure to infections.

For the LSCs within the BCR-ABLp210 transgenic mice, an oncogene-driven perturbation(s) to the epigenome appears to have set their lineage fate as to an altered DNA methylation patterns seen at the gene promoters within BCR-ABLp210 LSCs as compared to HSCs. In essence, these epigenetic lesions are reversible, and, therefore, repairable by the use of demethylating agents. The following studies have examined other epigenetic routes to therapies for cancer and to the provision of prognostic markers. Qin et al. have identified 114 microRNAs (miRNAs) that are disordered in hepatocellular carcinoma (HCC), providing some useful prognostic markers for HCC. For 93 of the miRNAs, their disorder is due to dysregulation of transcription factors (TFs), emphasizing the importance of the interplay between TFs and miRNAs. Seven up-regulated miRNAs can promote tumorigenesis, via inhibiting tumor suppressor genes. Seven down-regulated miRNAs can suppress the action of a number of oncogenes. Regarding targets for therapies, FOXO1 can activate tumor suppressor miRNAs. Ge et al. have reviewed the role of long noncoding RNA (lncRNA) in the epithelial-mesenchymal transition (EMT) in HCC. This transition, as driven by induced signals, transcription regulators, and their downstream effectors, plays a major role in tumor invasiveness and metastasis. A lncRNA-mediated positive feedback (signal) loop is conducive to the proliferation, differentiation, migration, and invasion of a wide variety of tumors. The loop is independent of the external environment, and regulates the process of EMT transition in HCC. LncRNAs act as RNA sponges, and in HCC to sequester inhibitory miRNAs that affect major signaling pathways (e.g., Wnt/β-catenin) that regulate the EMT of HCC. LncRNAs have also been observed to enhance drug (soratenib) resistance. These studies are largely at the basic research stage, but there is the hope that lncRNAs will find a clinical use.

From undertaking a pan-cancer analysis, Jin et al. have examined the possible oncogenic role(s) of the phosphorylated CTD-interacting factor 1 (PCIF1). PCIF1 is the only known methytransferase of N6,2′-O-dimethytransferase (m6Am) in mRNA. Expression was observed to be increased in most cancers as compared to normal tissue. High expression correlated positively with overall disease survival, disease-free survival, and the infiltration of CD4^+^ T cells in renal clear cell carcinoma and of CD8^+^ T-cells, macrophages, and B-cells in thyroid carcinoma. As to the infiltration, PCIF1 expression correlated positively with the expression of immune checkpoint genes. PCIF1 expression also correlated positively with tumor mutation burden (TMB) for some cancers (mesothelioma, lung adenocarcinoma, glioblastoma multiform, and lower grade glioma), and negatively for others (breast invasive carcinoma, colon adenocarcinoma, endometrial carcinoma, prostate adenocarcinoma, thymoma, and thyroid carcinoma). There were no overlaps for the correlations between the TMB and for the microsatellite groups. Whether PCIF1 plays a role in cancer to modify m6Am is as-yet unclear, and, as seen from the above, the potential activities of PCIF1 are varied.

A focus on the epigenome of normal versus cancer stem cells is a return to Conrad Waddington’s metaphor proposal for gene regulation, in 1957, of an epigenetic landscape that controls the lineage fate of developing stem cells. He envisaged that stem cells roll down various valleys with epigenetic events restricting (the hills to the valleys) the cell lineage fate of stem cells. Presently, there is still much more to learn about whether the epigenome is the “master orchestrator” of the cell lineage choice of stem cells, how some oncogenes might disrupt the landscape to set the fate of cancer stem cells, and how the later might be repaired to provide new treatments for cancer.

